# Lifetime medical costs of chronic hepatitis C in the United States

**DOI:** 10.1186/s12913-026-14360-1

**Published:** 2026-03-21

**Authors:** Taiwo O. Abimbola, Hasan Symum, Michelle Van Handel, Eyasu Teshale, William Thompson

**Affiliations:** 1 Office of the Director, National Center for HIV, Viral Hepatitis, STD, and TB Prevention, Centers for Disease Control and Prevention, Atlanta, Georgia USA; 2 Division of Viral Hepatitis, National Center for HIV, Viral Hepatitis, STD, and TB Prevention, Centers for Disease Control and Prevention, Atlanta, Georgia USA

**Keywords:** Hepatitis C, HCV, Antiviral agents, Costs, Cost analysis, Economic burden, Health care costs

## Abstract

**Background:**

More than 2.4 million people were estimated to have chronic hepatitis C (CHC) in the United States from 2017 to 2020. Direct-acting antiviral (DAA) therapy can cure hepatitis C and limit its complications, including liver cirrhosis and hepatocellular carcinoma (HCC). This study aims to estimate the lifetime medical cost burden of CHC in the United States.

**Methods:**

We estimated the lifetime direct medical costs of CHC by disease stage, using medical and laboratory claims data and a Markov transition model. The analysis considered the cost of CHC separately for persons treated with DAAs and untreated persons. We estimated the cost of health care utilization associated with CHC per person for the initial year, remaining life years, and overall lifetimes and used these costs to estimate lifetime costs for the 2020 cohort of adults with CHC in the United States in 2020.

**Results:**

On average, medical costs per person in a disease stage were $89,070 (treated) and $150,669 (untreated) for non-cirrhosis, $104,961 (treated) and $197,469 (untreated) for cirrhosis, $59,120 (treated) and $208,646 (untreated) for decompensated cirrhosis, $44,876 (treated) and $113,975 (untreated) for hepatocellular carcinoma. The overall per person lifetime medical cost (weighted by the percentage of newly reported cases in each disease stage) was $90,089 (treated) and $155,930 (untreated). For a cohort of 2.228 million people, the projected total medical cost burden of CHC in 2022 dollars would be $200 billion if all were treated using DAAs, compared to $347 billion if all were untreated.

**Conclusions:**

We found a substantial reduction in the overall lifetime medical costs of hepatitis C for persons treated with DAAs compared to untreated persons. These potential health care savings from expanded hepatitis C treatment underscore the importance of expanding HCV testing and DAA treatment as core components of the national strategy for hepatitis C elimination.

**Supplementary Information:**

The online version contains supplementary material available at 10.1186/s12913-026-14360-1.

## Background

Over 2.4 million adults in the United States were estimated to have hepatitis C during 2017–2020 [1, 2]. Untreated chronic hepatitis C (CHC) can lead to medical complications including liver cirrhosis, hepatocellular carcinoma, and liver transplantation. Eight to twelve weeks of treatment with direct-acting antivirals (DAA) can cure most patients, reducing progression of liver damage and averting liver-related deaths. Despite the availability of DAAs, thousands continue to die of complications from hepatitis C each year in the United States [3]. Many people with hepatitis C in the United States are unaware of their infection status and thus do not initiate treatment, and persons who know they are infected may not get treated for a variety of reasons, including the high price of treatment [4]. The *Viral Hepatitis National Strategic Plan for the United States: A Roadmap to Elimination (2021–2025)* outlines the core indicators needed to be met by 2030 to mitigate the national hepatitis C crisis. This includes reducing acute hepatitis C cases by 90%, increasing the proportion of people who have cleared hepatitis C to 80%, and reducing the rate of hepatitis C-related deaths by 65% [5]. These goals are largely dependent on increasing the number of persons diagnosed and treated for hepatitis C and the associated reductions in transmission, disease progression, and liver-related death.

Prior cost analyses on the burden of hepatitis C exist; however, most available studies on the costs of CHC rely on claims data from 2001 to 2012 before the availability and transition to DAA therapy [6–15]. Recent studies provide economic burden estimates for the Medicaid and Veterans’ Affairs (VA) populations, excluding the commercially insured population [16, 17]. Existing cost studies also do not reflect the shifting landscape of new infections driven by increased injection drug use and the estimated prevalence of hepatitis C adjusted to account for persons who inject drugs (~ 4.0 million) [18]. 

## Methods

We estimated lifetime direct medical costs of CHC in the United States for the prevalent cohort of adults aged ≥ 18 years in 2020. We modeled expected medical costs over the remaining lifetime using estimates of medical costs for DAA-treated and untreated people with CHC. Frequencies of medical visits and costs for inpatient and outpatient care related to CHC between 2015 and 2022 were derived from Merative MarketScan claims data. Marketscan is a healthcare utilization database that collects information on over 100 million people, comprised of commercially insured (employer-sponsored health insurance) for persons < 65 years and Medicare-eligible persons ≥ 65 years with Medicare supplemental insurance for a sub-set of Medicaid states.

We identified persons with CHC in Marketscan as the first positive hepatitis C virus (HCV) RNA test using the Logical Observation Identifiers Names and Codes (LOINC) or a hepatitis C diagnosis using International Classification of Diseases, 10th Revision, Clinical Modification (ICD-10-CM) codes (Table S1). Among persons with CHC, we defined a treated CHC case as a reported HCV DAA prescription in the MarketScan Pharmacy data after the diagnosis date (Table S2). If there was no reported HCV DAA code within 6 months of diagnosis, we defined those cases as untreated CHC cases. We assigned CHC cases to disease stage categories (non-cirrhosis, cirrhosis, decompensated cirrhosis (DC), hepatocellular carcinoma (HCC), and post liver transplant (PLT)). We extracted the inpatient and outpatient services records for any visit with HCV codes in MarketScan and estimated average cost by disease stage. Additional information on CHC case definitions by disease stage are described in the supplementary material. The inpatient or outpatient visit costs for each disease stage were calculated as the total payment reported in the Set B Marketscan data. The costs of DAA were derived from the MarketScan Redbook and adjusted for the regimen type used based on published estimates [19]. Additional information on deriving DAA regimen costs is available in the supplementary material. Due to the limited prevalence of claims for liver transplants in the MarketScan claims data, we relied on published estimates of the billed hospital and physician charges per liver transplant [20]. 

We developed a Markov transition model to estimate the medical cost of CHC per person by disease stage and over the lifetime of an average treated and an average untreated person. The direct medical costs were estimated using separate untreated and treated Markov transition models. Table 1 presents the costs and epidemiological inputs for hepatitis C used in the model.

**Table 1:** Markov transition model inputs

Inputs	Base value	Range	Source
**Costs in 2022 US dollars ($)**			
DAA	24,308	12,154–48,616	[19, 21]
2 HCV RNA tests**	394	187–729	[22]
*Treated costs*	*Outpatient; Inpatient*	*Range (Half – Double)*	[23]
Non-cirrhosis	93; 1458	47–186; 729–2916	
Cirrhosis	129; 4895	65–258; 2448–9790	
Decompensated cirrhosis	1323; 22,595	662–2646; 11,298–45,190	
Hepatocellular carcinoma	1568; 37,864	784–3136; 18,932–75,728	
*Untreated costs*	*Outpatient; Inpatient*	*Range (Half – Double)*	
Non-cirrhosis	134; 2916	67–268; 1458–5832	
Cirrhosis	116; 8285	58–232; 4143–16,570	
Decompensated cirrhosis	1593; 51,586	797–3186; 25,856–103,422	
Hepatocellular carcinoma	1954; 58,711	977–3908; 29,356–117,422	
Post Liver Transplant	1409; 64,619	705–2818; 32,310–129,238	
Liver transplant	592,896	296,488–1,185,792	[20]
**Age proportions**			
Age range	% Age*	*Not applicable*	[24]
18–19	0. 78		
20–29	17.5		
30–39	25.8		
40–49	15.7		
50–59	18.7		
60+	21.5		
**Annual reinfection rate**			
Reinfection rate	0.039	0.025–0.059	[25]
**Annual hospitalization rate**			
Treated HCV infection	0.082	0.00025-0.103	[26, 27]
Untreated non-cirrhosis	0.092	0.082–0.103	[27]
Untreated Cirrhosis	0.299	0.294–0.304	
Untreated DC	0.304	0.294–0.562	
Untreated HCC	0.304	0.294–0.562	
**Annual mortality rates for treated and untreated hepatitis C by stage**			
Treated non-cirrhosis and cirrhosis	*Mortality by age*		Table S4
Treated and Untreated DC	0.112	0.065–0.190	[28]
Treated and Untreated HCC	0.427	0.330–0.860	
**Annual transition rates for untreated chronic hepatitis C by stage**			
Non-cirrhosis to Cirrhosis***	0.037	0.033–0.040	[28, 29]
Cirrhosis to DC	0.039	0.010–0.079	
Cirrhosis to HCC	0.014	0.010–0.079	
DC to HCC	0.068	0.030–0.083	
DC to Liver transplant	0.04	0.000-0.140	
HCC to Liver transplant	0.023	0.010–0.062	

Direct-acting antivirals (DAA), Hepatitis C Virus (HCV), HCV Ribonucleic Acid (RNA), Decompensated Cirrhosis (DC), Hepatocellular Carcinoma (HCC)

* Proportion of reported chronic hepatitis C cases by age. **2 HCV RNA tests including one for hepatitis C diagnosis and one for cure. ***We calculated a single transition rate from f0-f3 to f4 = 0.037 using the fibrosis progression rates and distribution of persons with CHC by fibrosis stage from published literature [28, 29]. Further description of how we calculated the transition rate is provided in the supplementary material

### Treated CHC

A Markov transition model with disease stages (non-cirrhosis, cirrhosis, DC, and HCC) allowing reinfections was developed to represent persons who were treated for CHC (Supplementary Material, Figure S1). The model assumed 85% treatment success rate and that each person with CHC who was treated with DAAs at the start or originating disease stage remains in that disease stage in which they were treated and does not progress subsequently. Therefore, each person entered the treated model in an assigned health state and transitioned out of the model via death. Inpatient and outpatient costs were either defined as recurring annually (outpatient) or as an annual probability (hospitalization). Outpatient costs for treated persons were assigned on an annual basis for each health state using the average number of visits in MarketScan. Inpatient costs were adjusted for the hospitalization events each year by disease stage using published event rates for CHC [27]. The model assumed no progression for treated CHC but allowed for reinfection and retreatment. We simulated costs for 10,000 random persons over an analytic horizon starting in 2020 for 59 one-year cycles.

### Untreated CHC

Another Markov transition model was developed to represent persons with untreated CHC by disease stage (non-cirrhosis, cirrhosis, DC, HCC, post-liver transplant (PLT)) and death (Figure S2). Like the treated model, persons entered the model in an assigned health state and transitioned out of the model via death. Health care utilization costs were either defined as occurring annually (i.e., outpatient) or with a given probability (i.e., hospitalization). Unlike the treated model, this model allowed disease progression for untreated CHC. The probability of progression (i.e., transition) from one stage to the next for untreated CHC was derived from published literature [28, 29]. We simulated costs for 10,000 persons over an analytic horizon starting in 2020 for 59 one-year cycles.

### Estimating cost per person and cohort

Per person costs (including outpatient, inpatient, DAA treatment in the first year only, and HCV RNA testing) were estimated using three steps. In step 1, we calculated the initial year medical costs per person separately for treated and untreated CHC by disease stage. We summed the yearly outpatient and inpatient costs (weighted by the annual rate of hospitalization) per person. In step 2, we calculated the remaining life-year medical costs per person by disease stage separately for treated and untreated persons in the Markov transmission model. We then summed the initial year medical costs (claims data) and remaining lifetime medical cost (modeled projections) to derive the total lifetime medical cost per person separately for treated and untreated persons. We calculated the unadjusted cost for treated and untreated person by disease stage and adjusted costs using the percentage of persons with CHC by disease stage (Table S5). Lastly, in step 3, we extrapolated our results to 2 hypothetical cohorts of 2,228,000 (low estimate) and 4,043,200 (high estimate) adults with hepatitis C in the United States and calculated the projected savings [1, 2]. 

All costs were adjusted to 2022 USD using the annual price index of personal consumption expenditure which includes expenditures on behalf of consumers and third-party payers [30]. Future costs were discounted at an annual rate of 3%. The analysis excluded the cost of drugs and labs if not included as part of the inpatient or outpatient visit. We conducted one-way sensitivity analyses on costs (DAA, inpatient and outpatient) and epidemiological parameters (reinfection and hospitalization events rates) for both treated and untreated model scenarios. Furthermore, we explored the cost implications of increased disease progression in the treated model, assuming people treated with DAA would experience disease progression rates that were 50% of those assumed in the untreated model. The analysis was conducted in R (version 4.2.2) and TreeAge (version R2), 2022.

## Results

The average frequencies of inpatient and outpatient visits per person treated and untreated are shown in Table 2. The average annual outpatient visit frequencies were consistently lower by disease stage per person treated compared to untreated. Those in the non-cirrhosis stage had lower average annual outpatient visit frequency among the treated (0.13) and untreated (0.17) compared to those in more advanced stages. The HCC stage had the highest outpatient visits among the treated (2.05) and untreated (2.23). The average annual inpatient visits were lower in the non-cirrhosis stage among the treated (0.05) and untreated (0.10), and highest in the HCC state among the treated (0.89) and untreated (1.38). Table 2 also presents the average annual medical costs per person treated and untreated. The outpatient cost per person treated in the initial year ranged from $93 (non-cirrhosis) to $1,568 (HCC) and for the untreated ranged from $134 (non-cirrhosis) to $1,954 (HCC). For inpatient costs, the costs per person treated in the initial year ranged from $120 (non-cirrhosis) to $3,105 (HCC) and for the untreated ranged from $268 (non-cirrhosis) to $17,848 (HCC).

**Table 2:** Initial year medical visits and costs per person

Claims data						
Scenarios	Outpatient visits per year	Inpatient visits per year	Outpatient cost	Inpatient cost	DAA and HCV RNA test cost*	Average initial year cost
**Treated**						
Non-cirrhosis	0.13	0.05	$ 93	$ 120	$ 28,747	$ 28,959
Cirrhosis	0.19	0.13	$ 129	$ 401	$ 28,747	$ 29,277
DC	1.93	0.53	$ 1323	$ 1853	$ 28,747	$ 31,923
HCC	2.05	0.89	$ 1568	$ 3105	$ 28,747	$ 33,420
**Untreated**						
Non-cirrhosis	0.17	0.10	$ 134	$ 268	$ 394	$ 796
Cirrhosis	0.15	0.22	$ 116	$ 2477	$ 394	$ 2987
DC	1.96	1.21	$ 1593	$ 15,682	$ 394	$ 17,669
HCC	2.23	1.38	$ 1954	$ 17,848	$ 394	$ 20,196

*Redbook cost estimate for an assumed distribution of Direct acting antivirals (DAA) regimen – refer Table S3 for regimen types and prices, Hepatitis C Virus (HCV), Decompensated cirrhosis (DC), Hepatocellular Carcinoma (HCC)

*Two HCV RNA tests in the treated group for pretreatment, posttreatment assessment, and one HCV RNA test in the untreated group

Table 3 shows the average medical costs per person in the initial year, remaining lifetime, and overall. In the initial year, the average cost per person treated, incorporating the costs of outpatient visits, inpatient visits, DAAs, and two HCV RNA tests, ranged from $28,959 (non-cirrhosis) to $33,420 (HCC). The cost per person untreated in the initial year, incorporating the costs of outpatient visits, inpatient visits, and one HCV RNA test, ranged from $796 (non-cirrhosis) to $20,196 (HCC). The overall weighted cost per person was $29,040 (treated) and $1,283 (untreated) in the initial year.

The per person cost for the remaining and total lifetime based on modeled scenarios for the treated and untreated are shown in Table 3. The medical cost per person for the remaining lifetime by disease stage for non-cirrhosis was $60,111 (treated) and $149,873 (untreated), for cirrhosis was $75,684 (treated) and $194,482 (untreated), for DC was $27,197 (treated) and $190,977 (untreated), and for HCC was $11,456 (treated) and $93,779 (untreated). Medical costs per person by disease stage for the remaining life years tended to be higher in the earlier disease stages (i.e., non-cirrhosis, cirrhosis, and DC) due to a higher probability of survival compared with the HCC stage where over 90% would have died within the first 10 years (Figure S4 (panel A and B)). After adjusting for the percentage of persons with CHC by disease stage, the modeled remaining lifetime medical cost per person was $61,049 among those treated and $154,647 among those untreated. The medical costs per person overall and by hepatitis C liver stage derived by summing the initial year and remaining lifetime costs are presented in Table 3. The weighted total lifetime medical cost per person was $90,089 (treated) and $155,930 (untreated).

**Table 3:** Remaining lifetime medical cost (RLMC) and total lifetime medical cost (TLMC) per person

Scenarios	Claims data	Markov model		Overall
Treated	Average initial year cost	RLMC per person	95% prediction interval	TLMC per person*
Non-cirrhosis	$ 28,959	$ 60,111	59,369–60,852	$ 89,070
Cirrhosis	$ 29,277	$ 75,684	74,905–76,462	$ 104,961
DC	$ 31,923	$ 27,197	26,523–27,871	$ 59,120
HCC	$ 33,420	$ 11,456	11,072–11,839	$ 44,876
Weighted total	$ 29,040	$ 61,049	n/a	$ 90,089
**Untreated**				
Non-cirrhosis	$ 796	$ 149,873	146,137–153,610	$ 150,669
Cirrhosis	$ 2987	$ 194,482	190,287–198,678	$ 197,469
DC	$ 17,669	$ 190,977	186,388–195,566	$ 208,646
HCC	$ 20,196	$ 93,779	90,345–97,212	$ 113,975
Weighted total	$ 1283	$ 154,647	n/a	$ 155,930

*TLMC includes the RLMC and the average initial year costs. 95% prediction interval is not applicable (n/a) to the weighted costs. Direct-acting antivirals (DAA), Hepatitis C Virus (HCV), Decompensated cirrhosis (DC), Hepatocellular Carcinoma (HCC)

Applied to a hypothetical cohort of adults with hepatitis C in 2020 using the low hepatitis C prevalence estimate of 2,228,000 adults, the overall medical cost burden was $200 billion (treated) compared to $347 billion (untreated). However, if the hepatitis C prevalence was as high as 4,043,200 adults, the overall medical cost burden was $364 billion (treated) and $630 billion (untreated). Our estimates suggest that if the hepatitis C 2020 prevalent population were treated, $146 billion to $266 billion in discounted lifetime medical costs might be averted compared with if none of the prevalent population received curative treatment: a cost savings of approximately $65,841 per person.

**Fig. 1:**
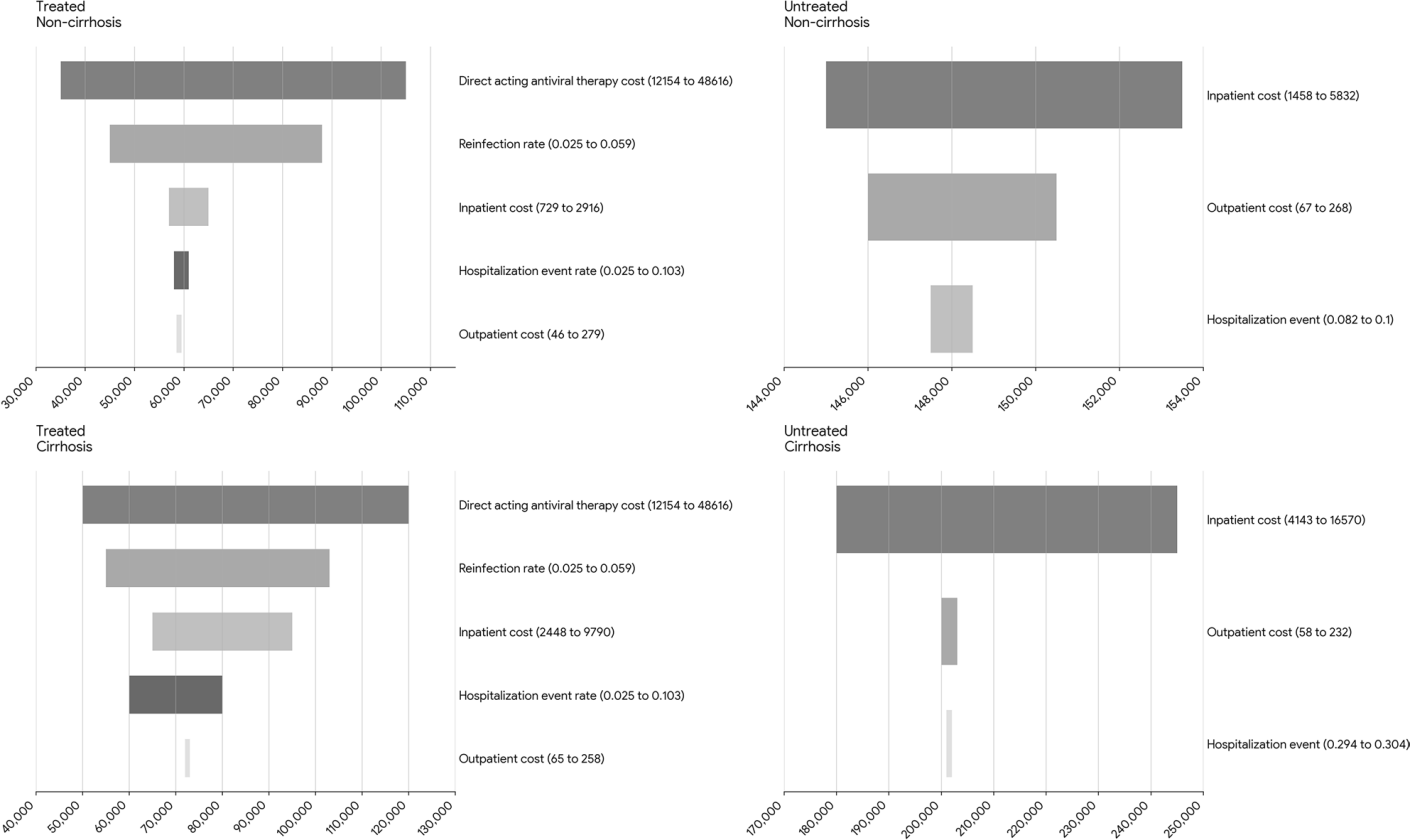
Sensitivity analysis – Varying remaining lifetime medical costs of chronic hepatitis C. * Direct-acting antivirals (DAA), Hepatitis C Virus (HCV)

**Fig. 2:**
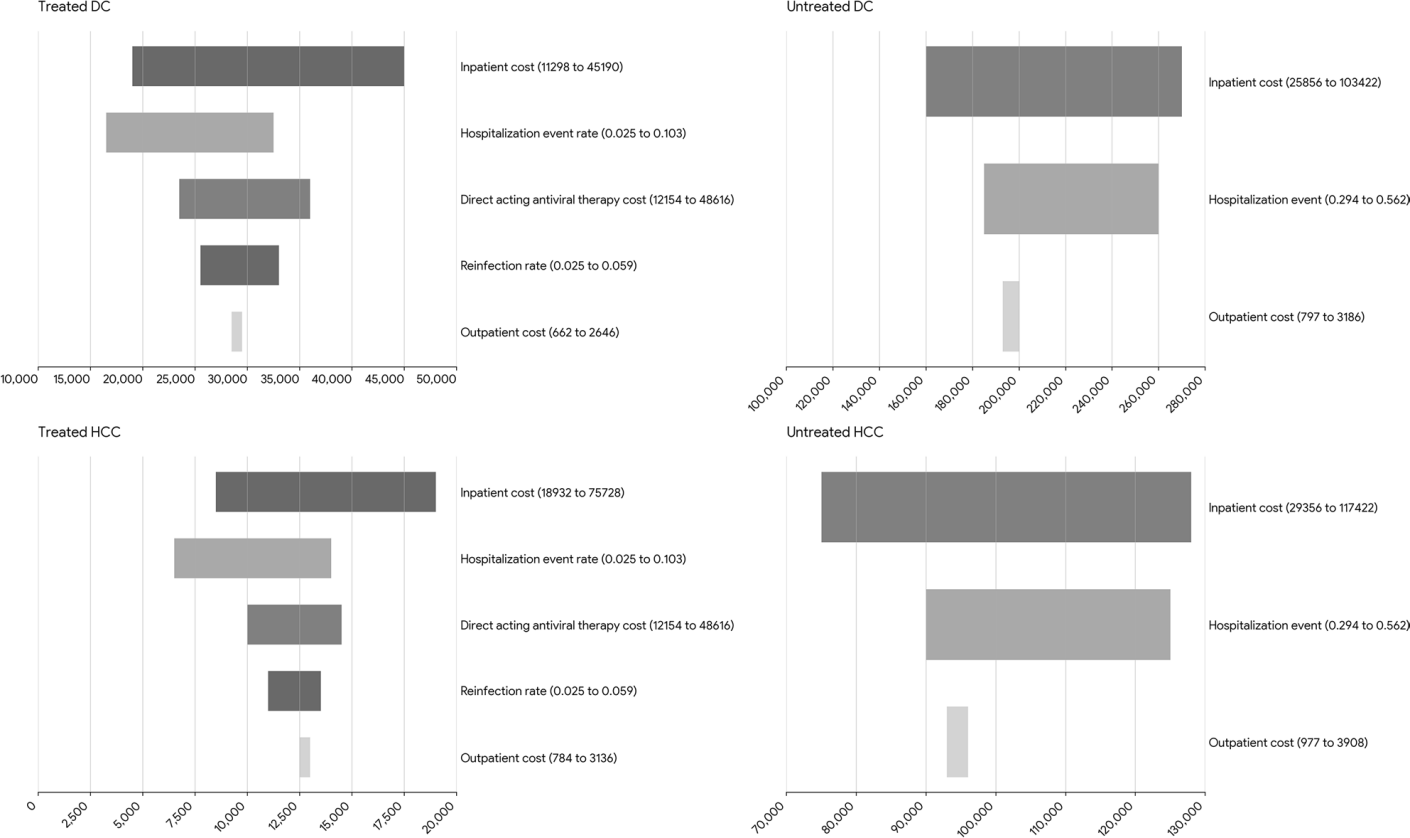
Sensitivity analysis – Varying the remaining lifetime medical costs of chronic hepatitis C. * Direct-acting antivirals (DAA), Hepatitis C Virus (HCV), Decompensated cirrhosis (DC), Hepatocellular Carcinoma (HCC)

The results of the bivariate sensitivity analyses for specific modeled parameters are shown in Figs. 1 and 2. Figure 1 shows that DAA costs and HCV reinfection rates largely account for the widest variation in costs per person treated in the earlier disease stages (cirrhosis and non-cirrhosis), while inpatient costs drove the variation in costs per person in the earlier disease stages for untreated persons. Figure 2 presents findings from the sensitivity analysis for those in the later disease stages. The annual cost per inpatient visit and the annual rate of hospitalization produced the widest variation in costs for HCC and DC. Further sensitivity analysis of the treated model assuming disease progression rates that were about half of that for untreated persons showed a 3% increase in the average lifetime medical per person treated.

## Discussion

Our study estimated the lifetime direct medical cost of CHC per person treated ($90,089) and untreated ($155,930). This finding is partly explained by the differences in health care utilization per person and the costs accrued over the lifetime per person treated compared to untreated. The inpatient visit utilization per person was lowest at each stage for those treated compared to untreated. Overall medical costs were consistently higher after the initial year for the untreated compared to those treated with DAAs, capturing continuing disease progression and the associated ongoing CHC-related health care utilization among untreated persons.

A Medicaid study estimated the average hepatitis C cost per person per year in 2020 at $20,000 (2022 USD) [16]. A VA study estimated a total lifetime cost per person at $199,792 treated and $262,000 untreated (in 2021 USD) [17]. The VA study estimated a total projected lifetime cost savings per person treated of $77,484 (including DAA price at $21,905)compared to our estimate of $65,841 (including DAA price at $24,308). The mean age for patients in the VA study was 63 years while the mean age in our study was 49 years (Figure S3).

Even though an estimated 1.2 million people in the United States with hepatitis C were treated during 2014–2020, many with hepatitis C are unaware of their infection [19, 31]. This lack of awareness coupled with ample missed opportunities to diagnose, cure, and prevent subsequent transmission of hepatitis C, continues to hinder progress towards hepatitis C elimination in the United States. One analysis of timely DAA treatment (i.e., defined as within 12 months) among insured persons diagnosed with hepatitis C, found treatment was associated with type of insurance coverage; 35% of persons with private insurance initiated treatment compared with 23% of those with Medicaid during 2019–2020 [32]. Significant financial investments are needed to ensure all persons with hepatitis C are diagnosed, linked to care, treated, and cured. Because we did not quantify the extent of the financial investments needed to detect incident cases of hepatitis C, our findings of cost savings from treatment should not be interpreted as representing the cost needed to reach elimination.

Our analysis has several limitations. We use a Markov transition model to estimate the cost of CHC, which assumes a closed cohort and does not allow transmission dynamics beyond the static cohort. This underestimates the savings to society when the secondary benefits of treatment are not included. The modeled treatment scenario is a counterfactual scenario and does not portray the status quo in United States representing close to 1.2 million people with HCV treated between 2014 and 2020. In addition, our model does not account for demographic characteristics of persons with CHC such as sex or behaviors like injection drug use, so we are not able to report differences in lifetime medical cost of CHC for males versus females or for those at a higher risk of HCV reinfection. We assumed those who became reinfected remained in the same disease stage (after retreatment) as when they initiated DAA treatment for simplicity of the treated model which likely underestimated costs. Reinfection can lead to disease progression; and is more likely to occur in cases among people who inject drugs [33].

DAAs do not directly treat existing liver damage but are associated with slower progression to HCC overall [34]. While our main treated scenario assumed no progression, some people might still progress to later stages of disease even with treatment. Further exploration of increased disease progression among those treated with DAA in our model showed a modest 3% increase in the average lifetime medical per person treated. Our model does not incorporate age-specific fibrosis progression rates, which may bias estimates of advanced liver disease (understating costs in older adults and overstating it in younger ones). Because disease stage strongly affects lifetime medical costs, this limitation may influence long‑term cost estimates. We partially addressed this by including age‑stratified mortality data, though it does not fully capture age‑specific disease progression. In addition to liver cirrhosis and HCC, many hepatitis C-associated extrahepatic manifestations, including cardiovascular, neurological, metabolic (i.e., steatotic liver disease and alcohol-related liver disease), and renal conditions, play a role in increasing HCV morbidity and mortality [35, 36]. Antiviral therapy is associated with reductions in mortality due to HCV-associated extrahepatic manifestations [37–39]. An economic analysis of hepatitis C showed cost savings due to reductions in extrahepatic conditions such as diabetes and chronic kidney disease resulting from DAA treatment [6]. We did not include the impact of these extrahepatic manifestations which could have underestimated the projected cost savings from DAA treatment; however, our analysis included all costs included with the hepatitis C patient visit and potentially captures some costs associated with extrahepatic manifestations.

Our analysis uses total payments related to inpatient and outpatient visits for hepatitis C. Total payments reflect insurer’s allowed charges and do not reflect the actual provider costs for each visit, which could be lower than reported charges. Furthermore, our analysis excludes aspects of the full economic burden of CHC such as out-of-pocket expenditures (e.g., co-payments for other drugs and lab tests, transportation, and care-giving costs) and indirect costs (i.e., productivity loss and intangible costs related to pain and suffering), thereby underestimating the full economic burden of CHC in the United States.

## Conclusions

We quantified the medical cost of CHC in the United States and showed there is substantial ongoing health care utilization among persons who remain untreated resulting in higher lifetime medical costs. Disproportionately higher medical costs among untreated persons could be greatly lowered if they were treated as demonstrated by the lower costs among persons treated with DAAs. Our findings underscore the potential savings from expanded hepatitis C treatment as a core component hepatitis C elimination in the United States.

## Supplementary Information

Below is the link to the electronic supplementary material.

Supplementary Material 1

## Data Availability

The dataset supporting the conclusion of this article is available upon reasonable request from the corresponding author.

## Abbreviations

DAA: Direct-Acting Antiviral
DC: Decompensated Cirrhosis
PLT: Post Liver Transplant
CHC: Chronic Hepatitis C
HCC: Hepatocellular Carcinoma
HCV: Hepatitis C Virus
RLMC: Remaining Lifetime Medical Cost
RNA: Ribonucleic Acid
TLMC: Total Lifetime Medical Cost
