# Supplementary material for: Lifetime medical costs of chronic hepatitis C in the United States
Source: BMC Health Serv Res. 2026 Mar 21;26:609. doi: 10.1186/s12913-026-14360-1 (PMC13126785; doi:10.1186/s12913-026-14360-1)
Supplement: Supplementary file 1 — Supplementary Material 1 [file 12913_2026_14360_MOESM1_ESM.docx]

**Supplementary Material**

**Lifetime medical costs of chronic hepatitis C in the United States**

This supplementary material provides additional information on the data sources and model used in this study. Information regarding relevant model parameters, assumptions, and results are presented in the main text.

Contents

[Section 1: Model inputs 2](#_Toc216077265)

[Section 2: Model set-up and estimation 5](#_Toc216077266)

[Section 3: Model outputs 9](#_Toc216077267)

[References 13](#_Toc216077268)

# Section 1: Model inputs

Disease stage categorization using Marketscan

Laboratory test and clinical observation codes (Logical Observation Identifiers Names and Codes - LONIC) and the chronic hepatitis C diagnosis code (ICD-10) and were used to identify inpatient and outpatient visit records in Marketscan. We applied the following hepatitis C disease stage categories to data in Marketscan using the ICD-10 and LOINC provided in Table S1 below.

- Non-cirrhosis: HCV case identified by positive HCV RNA test and FIB-4 <3.25 (with no DC, HCC and liver transplant (LT) diagnosis in same month).
- Cirrhosis: HCV case identified by positive HCV RNA test and FIB-4 >=3.25 (with no DC, HCC and LT diagnosis in same month).
- Decompensated cirrhosis: Any DC ICD 10 diagnosis claims in inpatient settings.
- Hepatocellular Carcinoma: Any HCC ICD 10 diagnosis claims in inpatient settings.
- Liver Transplant: Any liver transplant ICD 10 diagnosis claims in inpatient settings.

**Table S1:** Hepatitis C ICD-10-CM and LOINC codes (Isenhour, 2018)

| Case category | Code (ICD 10/ LOINC code) |
| --- | --- |
| Chronic Hepatitis C | Diagnosis code: B18.2 |
| HCV RNA Test | LOINC code: 758862,139550,161281,161299,169367,223248,223255,223263,223271,223289,223297,407262,425066,448316,473652, 473652,474411,481598,516567,516575,518241,51987,533760,570069,723767, 758888,106765,110114,112599,204164,205716,296095,347039,347047,381806,389981,420034,426171,472522,485763,493692,493700,493726,493734,493742,493759,493767,493775,493783,493791,493809,496034,496042,496059,496083,49758,500231,50104,50112,50120,516559,322867 |
| Non-cirrhosis | LOINC 98491-4, 98488-0 |
| Cirrhosis | LOINC 98491-4, 98488-0; ICD 10 codes: K74.3, K74.4, K74.5, K74.60, K74.69 |
| Decompensated cirrhosis (DC) | R18.0, R18.8, K76.6, K65.0, K65.1, K65.2, K65.8, K65.9, I85.00, I85.01 |
| Hepatocellular carcinoma (HCC) | C22.0 |
| Liver Transplant (LT) | Z944, 0FY00Z0, 0FY00Z1 |

We defined a treated CHC case as an individual with a reported HCV DAA prescription in the MarketScan Pharmacy data using the HCV DAA National Drug Codes (NDC) in Table S2.

**Table S2:** DAA NDC codes (Auty, 2021)

| **Drug name** | **Active Ingredients** | **11-Digit NDC** |
| --- | --- | --- |
| Sovaldi | Sofosbuvir | 61958150101 |
| Sovaldi | Sofosbuvir | 61958150301 |
| Sovaldi | Sofosbuvir | 61958150401 |
| Sovaldi | Sofosbuvir | 61958150501 |
| Harvoni | Ledipasvir/sofosbuvir | 61958180501 |
| Harvoni | Ledipasvir/sofosbuvir | 61958180401 |
| Harvoni | Ledipasvir/sofosbuvir | 61958180301 |
| Harvoni | Ledipasvir/sofosbuvir | 61958180101 |
| Viekira pak | Ombitasvir/paritaprevir/ritonavir/dasabuvir | 00074309328 |
| Technivie | Ombitasvir/paritaprevir/ritonavir | 00074308228 |
| Zepatier | Elbasvir/grazoprevir | 00006307402 |
| Zepatier | Elbasvir/grazoprevir | 00006307401 |
| Epclusa | Sofosbuvir/velpatasvir | 61958220101 |
| Epclusa | Sofosbuvir/velpatasvir | 61958220301 |
| Epclusa | Sofosbuvir/velpatasvir | 61958220401 |
| Epclusa | Sofosbuvir/velpatasvir | 61958220402 |
| Epclusa | Sofosbuvir/velpatasvir | 61958220501 |
| Viekira XR | Dasabuvir/ombitasvir/paritaprevir/ritonavir | 00074006328 |
| Vosevi | Sofosbuvir/velpatasvir/voxilaprevir | 61958240101 |
| Mavyret | Glecaprevir/pibrentasvir | 00074262528 |
| Mavyret | Glecaprevir/pibrentasvir | 00074262580 |
| Mavyret | Glecaprevir/pibrentasvir | 00074260028 |
| Mavyret | Glecaprevir/pibrentasvir | 00074262584 |
| Mavyret | Glecaprevir/pibrentasvir | 00074308228 |
| Incivek | Telaprevir | 51167010001 |
| Incivek | Telaprevir | 51167010003 |
| Olysio | Simeprevir sodium | 59676022528 |
| Victrelis | Boceprevir | 00085031402 |
| Daklinza | Daclatasvir dihydrochloride | 0003001101 |
| Daklinza | Daclatasvir dihydrochloride | 0003021301 |
| Daklinza | Daclatasvir dihydrochloride | 0003021501 |

Cost data

We used the MarketScan S visit level file for inpatient claims, O service level file for outpatient claims, and the total payments (Set B data) in claims table S and O reporting actual financials for the cost data elements. These files were used to estimate the average cost for each year by disease stage cost for 1 year following the diagnosis index date and then compiled costs for 2015 through 2022 by inpatient (see ‘a’ below) and outpatient (see ‘b’ below) annual (one-year) costs:

1. Average number of hospital visits in one year multiplied by the average hospital payment per inpatient visit.
2. Average number of outpatient visits in one year multiplied by the average payment per outpatient visit.

DAA costs were derived using the MarketScan Redbook prices for regimen types from Teshale, 2022 (Table S3). Regimen types that were not included in the top 5 were combined into the “others” category and imputed with the highest price regimen type. We then calculated the average DAA regimen cost using a weighted price across the regimen types.

**Table S3:** Direct-acting antiviral regimen types, prices (Redbook), and percentages (Teshale 2022)

| Regimen types in Micromedex Redbook | 2023 Average wholesale prices | Percentatge using regimen type |
| --- | --- | --- |
| Epclusa (sofosbuvir + velpatasvir) | 28800 | 0.458 |
| Harvoni (ledipasvir + sofosbuvir) | 43200 | 0.065 |
| Mavyret (glecaprevir + pibrentasvir) | 15840 | 0.443 |
| Vosevi (sofosbuvir + velpatasvir + voxilaprevir) | 28800 | 0.004 |
| Zepatier (elbasvir + grazoprevir) | 26208 | 0.007 |
| Others+ SOF+/RIB+/DAK+/SIM+ | 43200 | 0.023 |
| Weighted average (by proportion using regimen type) | 24,308 |  |

# Section 2: Model set-up and estimation

The Markov state transition model was built using TreeAge Pro 2023, R1 Healthcare (TreeAge Software, Williamstown, MA).

**Figure S1:** Schematic of treated chronic hepatitis C progression by disease stage.


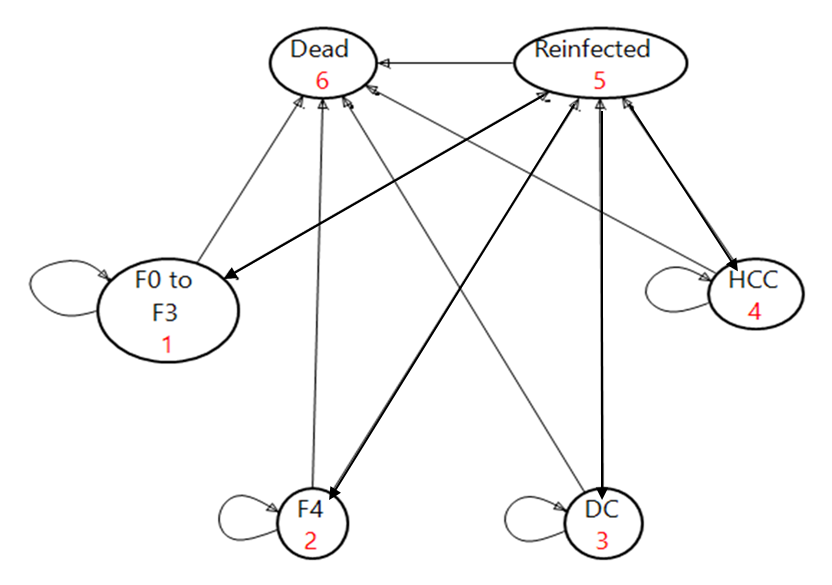


Fibrosis stage 0, 1, 2, and 3 (F0-F3= Non-cirrhosis); Fibrosis stage 4 (F4 = Cirrhosis); DC = Decompensated cirrhosis; HCC = Hepatocellular carcinoma). Reinfected persons remain in the original disease stage after treatment.

**Figure S2:** Schematic of untreated chronic hepatitis C progression by disease stage.


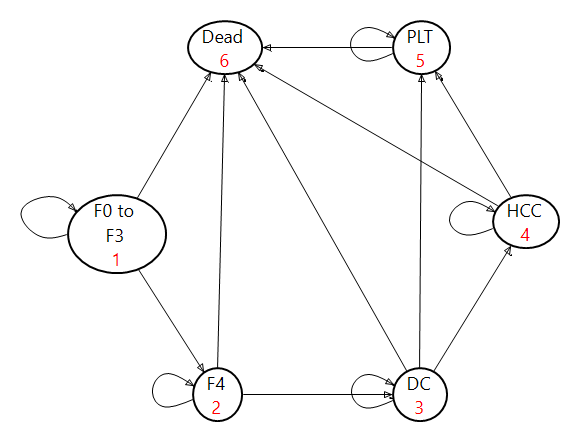


cirrhosis; HC

Fibrosis stage 0, 1, 2, and 3 (F0-F3= Non-cirrhosis); Fibrosis stage 4 (F4 = Cirrhosis); DC = Decompensated cirrhosis; HCC = Hepatocellular carcinoma; PLT = Post Liver Transplant).

Methods for treated and untreated chronic hepatitis C (CHC) calculation

Treated CHC

We developed a Markov transition model to represent individuals with treated for CHC using DAAs. The model defined disease stages (non-cirrhosis, cirrhosis, DC, and HCC) and three jump states (remained in disease stage/not reinfected, remained in disease stage/reinfected, and died). Individuals were stratified by age (Figure S3). The model assumes that each person with CHC is treated with direct acting antivirals (DAA) at the starting or originating disease stage and that no disease progression is expected with treatment (i.e., each individual stays assigned to the originating disease stage throughout the analytic horizon of the model). Therefore, individuals entered the model in an assigned disease stage and transitioned out of the model via death and can be reinfected/retreated. The probability of death was estimated using an age-adjusted background mortality probability for non-cirrhosis and cirrhosis (Table S4) and stage-specific transition to mortality for DC and HCC (Chhatwal, 2016). A transition flow diagram for the treated CHC model is depicted in Figure S1.

Modeled costs were either defined as recurring annually (outpatient costs) or as an event (inpatient costs). Outpatient costs were assigned on an annual basis to each disease stage using the average frequencies of patient visits from MarketScan. Inpatient costs were assigned based on the mean frequencies of patient hospitalizations adjusted for the hospitalization event rate in a given year by disease stage, using published event rates for CHC (Teshale, 2016).

The model was run separately for each disease stage to calculate costs on an annual and cumulative basis. For example, to calculate the lifetime costs for an individual starting in the non-cirrhosis stage (F0-F3), the model was run with an initial probability equal to 1 at the non-cirrhosis stage with other stages set to zero. The results for each disease stage were run using Monte Carlo simulation for 10,000 random walks over an analytic horizon of 0 to 59 one-year cycles.

Untreated CHC

We developed a Markov transition model to represent disease stages (non-cirrhosis, cirrhosis, DC, HCC, post liver transplant, and death) and jump states (remained in disease stage, progressed, and died) for individuals with untreated CHC. Disease progression was defined by transition probabilities (Table 1) representing each disease stage. Transition to death was assigned using age-adjusted background mortality probabilities for the non-cirrhosis and cirrhosis stages (Table S4) and stage-specific transition to mortality for DC and HCC (Chhatwal, 2016). A transition flow diagram for the untreated CHC model is depicted in Figure S2.

We calculated a single annual transition from non-cirrhosis (f0-f3) to cirrhosis (f4) as the probability of transition to f4 from each stage f0, f1, f2, and f3 weighted by the proportion in stages f0-f3. We used the following fibrosis progression rates from Erman et al., 2019 (f0=0.107, f1=0.082, f2=0.117, and f3=0.116), and assumed 89% of persons with CHC in stages f0-f3 are distributed as f0 (31%), f1(38%), f2(19%), and f3(12%) based on Chhatwal et al., 2016. We calculated the sum of transitions for f0-f3 = 0.0370 (i.e., 1/0.107 + 1/0.082 + 1/0.117 + 1/0.116), adjusted for the fibrosis stage distributions.

An initial probability of hospitalization (event) was used to calculate annual rewards (i.e., costs) for each stage. The costs in each disease stage were either defined as recurring annually (outpatient costs) or as an event (inpatient costs). Outpatient costs for individuals were assigned on an annual basis to each stage using the average frequencies of patient visits from MarketScan. Inpatient costs were also assigned as based on the mean frequencies of patient hospitalizations adjusted for the hospitalization event rate in a given year by disease stage, using published event rates for CHC (Teshale, 2016).

The model was run separately for each disease stage to calculate costs on an annual and cumulative basis. For example, to calculate the lifetime costs for an individual starting in the non-cirrhosis stage (F0-F3), the model was run with an initial probability equal to 1 at the non-cirrhosis stage with other stages set to zero. The results were run using Monte Carlo simulation for 10,000 random walks over an analytic horizon of 0 to 59 one-year cycles.

Other model inputs

The percentage of reported CHC cases by age were calculated by dividing the number of cases reported in each age range by the total number of CHC cases reported in 2019. For ages 18-19 years, we used the same number of cases reported for ages 0-19 years, assuming the majority of CHC cases reported are among older adolescents (Viral Hepatitis Surveillance Report, 2019).

**Figure S3:** Percentage distribution of chronic hepatitis C percent distribution by age used in Monte Carlo simulation.


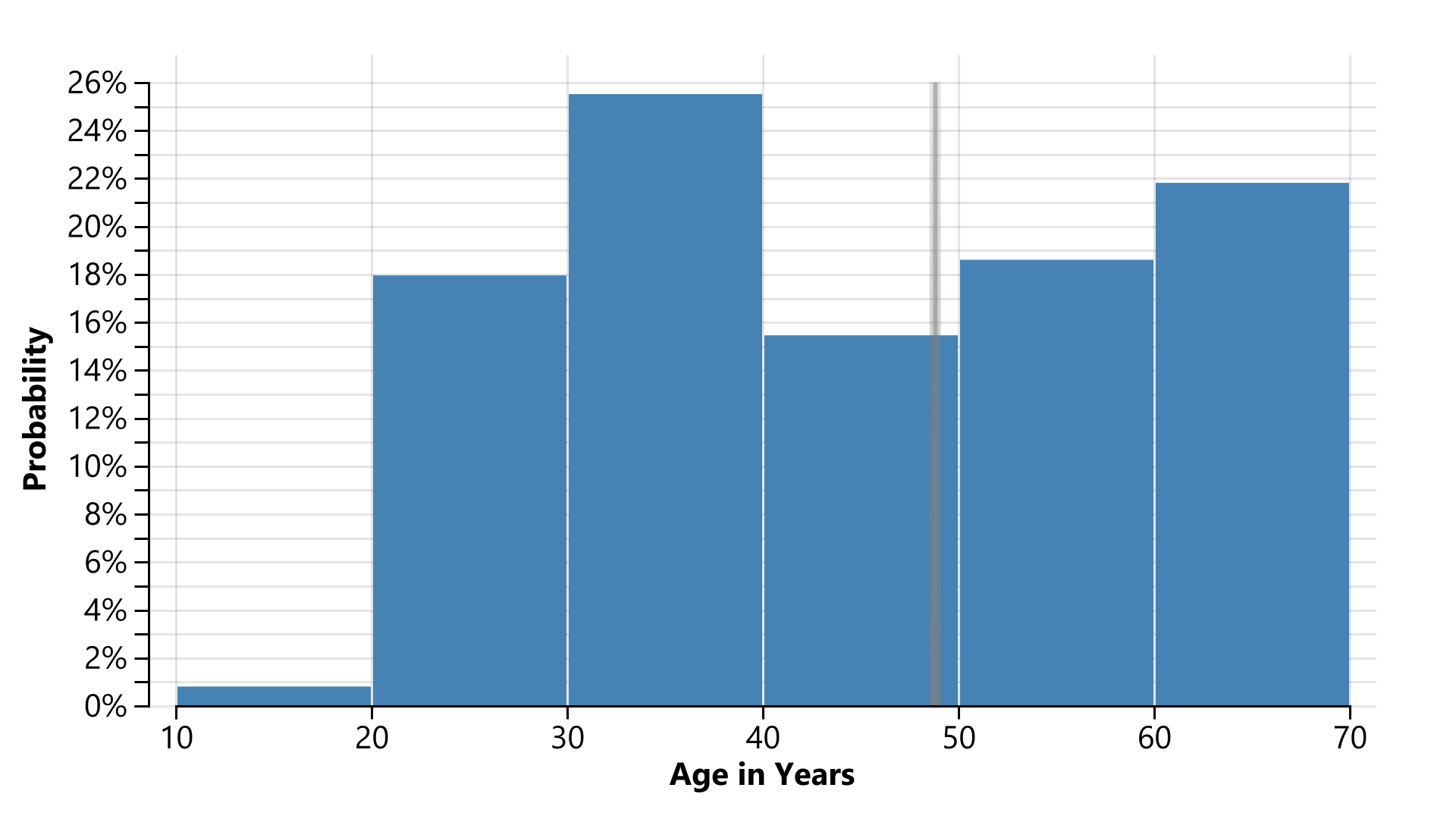


**Table S4:** National Vital Statistics System life table for total population, United States (2019).

| Age | Rate mortality |
| --- | --- |
| 18 | 6.87E-04 |
| 19 | 7.93E-04 |
| 20 | 9.03E-04 |
| 21 | 0.001011842 |
| 22 | 0.001106393 |
| 23 | 0.00117957 |
| 24 | 0.001236444 |
| 25 | 0.001286085 |
| 26 | 0.001338138 |
| 27 | 0.001395923 |
| 28 | 0.00146486 |
| 29 | 0.001543339 |
| 30 | 0.001626156 |
| 31 | 0.001707776 |
| 32 | 0.001788245 |
| 33 | 0.001865279 |
| 34 | 0.001940784 |
| 35 | 0.002023262 |
| 36 | 0.002112978 |
| 37 | 0.002202468 |
| 38 | 0.002290967 |
| 39 | 0.002383792 |
| 40 | 0.002490663 |
| 41 | 0.002616708 |
| 42 | 0.002759531 |
| 43 | 0.00291949 |
| 44 | 0.003098503 |
| 45 | 0.003303726 |
| 46 | 0.003536985 |
| 47 | 0.003794006 |
| 48 | 0.004075573 |
| 49 | 0.004387044 |
| 50 | 0.004720342 |
| 51 | 0.005093527 |
| 52 | 0.005532597 |
| 53 | 0.00604269 |
| 54 | 0.006602854 |
| 55 | 0.007168843 |
| 56 | 0.007743909 |
| 57 | 0.008372108 |
| 58 | 0.009074019 |
| 59 | 0.009841512 |
| 60 | 0.010669791 |
| 61 | 0.011514576 |
| 62 | 0.012344703 |
| 63 | 0.013146344 |
| 64 | 0.0139568 |
| 65 | 0.014819253 |
| 66 | 0.015850633 |
| 67 | 0.016977763 |
| 68 | 0.018272245 |
| 69+ | 0.019676199 |

**Table S5:** Percentage CHC by disease stage (Chhatwal, 2016).

| Disease stage | Percent (%) in stage |
| --- | --- |
| Non-cirrhosis | 88.78% |
| Cirrhosis | 9.61% |
| DC | 1.43% |
| HCC | 0.18% |

We summed the percentages for fibrosis stages F0, F1, F2, and F3 to derive the percentages in the non-cirrhosis stage using patient characteristics (Chhatwal, 2016). This information was used to weight the overall costs presented in the main results table 3 for treated and untreated chronic hepatitis C.

# Section 3: Model outputs

**Table S6:** Non-cirrhosis stage cycle proportions.

| Cycle | Non-cirrhosis | Cirrhosis | DC | HCC | Dead | LT |
| --- | --- | --- | --- | --- | --- | --- |
| 0 | 100% | 0% | 0% | 0% | 0% | 0% |
| 1 | 95% | 5% | 0% | 0% | 0% | 0% |
| 2 | 91% | 9% | 0% | 0% | 0% | 0% |
| 3 | 87% | 12% | 0% | 0% | 0% | 0% |
| 4 | 83% | 16% | 1% | 0% | 0% | 0% |
| 5 | 79% | 19% | 1% | 0% | 1% | 0% |
| 6 | 75% | 21% | 2% | 1% | 1% | 0% |
| 7 | 72% | 24% | 2% | 1% | 2% | 0% |
| 8 | 68% | 26% | 3% | 1% | 2% | 0% |
| 9 | 65% | 27% | 3% | 1% | 3% | 0% |
| 10 | 62% | 29% | 4% | 1% | 4% | 0% |
| 11 | 59% | 30% | 4% | 1% | 5% | 1% |
| 12 | 56% | 31% | 4% | 1% | 6% | 1% |
| 13 | 54% | 32% | 5% | 1% | 7% | 1% |
| 14 | 51% | 33% | 5% | 2% | 8% | 1% |
| 15 | 49% | 33% | 5% | 2% | 10% | 1% |
| 16 | 46% | 34% | 6% | 2% | 11% | 1% |
| 17 | 44% | 34% | 6% | 2% | 13% | 2% |
| 18 | 42% | 34% | 6% | 2% | 14% | 2% |
| 19 | 40% | 34% | 6% | 2% | 16% | 2% |
| 20 | 38% | 34% | 6% | 2% | 17% | 2% |
| 21 | 36% | 34% | 6% | 2% | 19% | 2% |
| 22 | 35% | 34% | 6% | 2% | 20% | 3% |
| 23 | 33% | 34% | 6% | 2% | 22% | 3% |
| 24 | 31% | 33% | 6% | 2% | 24% | 3% |
| 25 | 30% | 33% | 7% | 2% | 25% | 3% |
| 26 | 28% | 33% | 6% | 2% | 27% | 4% |
| 27 | 27% | 32% | 6% | 2% | 29% | 4% |
| 28 | 26% | 31% | 6% | 2% | 30% | 4% |
| 29 | 24% | 31% | 6% | 2% | 32% | 4% |
| 30 | 23% | 30% | 6% | 2% | 34% | 4% |
| 31 | 22% | 30% | 6% | 2% | 35% | 5% |
| 32 | 21% | 29% | 6% | 2% | 37% | 5% |
| 33 | 20% | 28% | 6% | 2% | 39% | 5% |
| 34 | 19% | 28% | 6% | 2% | 40% | 5% |
| 35 | 18% | 27% | 6% | 2% | 42% | 6% |
| 36 | 17% | 26% | 6% | 2% | 44% | 6% |
| 37 | 16% | 25% | 6% | 2% | 45% | 6% |
| 38 | 15% | 25% | 5% | 2% | 47% | 6% |
| 39 | 15% | 24% | 5% | 2% | 48% | 6% |
| 40 | 14% | 23% | 5% | 2% | 50% | 7% |
| 41 | 13% | 22% | 5% | 1% | 51% | 7% |
| 42 | 12% | 22% | 5% | 1% | 53% | 7% |
| 43 | 12% | 21% | 5% | 1% | 54% | 7% |
| 44 | 11% | 20% | 5% | 1% | 56% | 7% |
| 45 | 10% | 19% | 5% | 1% | 57% | 7% |
| 46 | 10% | 19% | 4% | 1% | 58% | 8% |
| 47 | 9% | 18% | 4% | 1% | 60% | 8% |
| 48 | 9% | 17% | 4% | 1% | 61% | 8% |
| 49 | 8% | 16% | 4% | 1% | 62% | 8% |
| 50 | 8% | 16% | 4% | 1% | 63% | 8% |
| 51 | 7% | 15% | 4% | 1% | 65% | 8% |
| 52 | 7% | 14% | 4% | 1% | 66% | 8% |
| 53 | 6% | 14% | 3% | 1% | 67% | 8% |
| 54 | 6% | 13% | 3% | 1% | 68% | 9% |
| 55 | 6% | 12% | 3% | 1% | 69% | 9% |
| 56 | 5% | 12% | 3% | 1% | 70% | 9% |
| 57 | 5% | 11% | 3% | 1% | 72% | 9% |
| 58 | 5% | 10% | 3% | 1% | 73% | 9% |
| 59 | 4% | 10% | 3% | 1% | 74% | 9% |

**Figure S4:** CHC cases surviving by year

Panel A*

*Non-cirrhosis curve was offset by 10% from the primary axis to separate overlap with the Cirrhosis curve.

Panel B

# References

Auty SG, Shafer PR, Dusetzina SB, Griffith KN. Association of Medicaid Managed Care Drug Carve Outs With Hepatitis C Virus Prescription Use. JAMA Health Forum. 2021;2(8):e212285.

Chhatwal J, Wang X, Ayer T, Kabiri M, Chung RT, Hur C, et al. Hepatitis C Disease Burden in the United States in the era of oral direct-acting antivirals. Hepatology. 2016;64(5):1442-50.

Chhatwal J, Chen Q, Aggarwal R. Estimation of Hepatitis C Disease Burden and Budget Impact of Treatment Using Health Economic Modeling. Infect Dis Clin North Am. 2018;32(2):461-80.

Isenhour C, Hariri S, Vellozzi C. Monitoring the hepatitis C care cascade using administrative claims data. Am J Manag Care. 2018 May;24(5):232-238. PMID: 29851440; PMCID: PMC6371394.

Teshale EH, Xing J, Moorman A, Holmberg SD, Spradling PR, Gordon SC, et al. Higher all-cause hospitalization among patients with chronic hepatitis C: the Chronic Hepatitis Cohort Study (CHeCS), 2006-2013. J Viral Hepat. 2016;23(10):748-54.

Teshale EH, Roberts H, Gupta N, Jiles R. Characteristics of Persons Treated for Hepatitis C Using National Pharmacy Claims Data, United States, 2014-2020. Clin Infect Dis. 2022;75(6):1078-80.

Viral Hepatitis Surveillance Report. Number and rates of newly reported cases of chronic hepatitis C virus infection, by demographic characteristics — United States, 2019.
